# Generation of Insulin-Producing Cells from Canine Bone Marrow-Derived Mesenchymal Stem Cells: A Preliminary Study

**DOI:** 10.3390/vetsci11080380

**Published:** 2024-08-18

**Authors:** Antonella Colella, Giuseppina Biondi, Nicola Marrano, Edda Francioso, Laura Fracassi, Alberto M. Crovace, Alessandra Recchia, Annalisa Natalicchio, Paola Paradies

**Affiliations:** 1 Department of Precision and Regenerative Medicine and Ionian Area (DiMePre-J), Veterinary Clinics and Animal Production Section, University of Bari Aldo Moro, Valenzano, 70010 Bari, Italy; antonella.colella@uniba.it (A.C.); edda.francioso@uniba.it (E.F.); laura.fracassi@uniba.it (L.F.); alessandra.recchia1@uniba.it (A.R.); 2 Department of Precision and Regenerative Medicine and Ionian Area (DiMePre-J), Internal Medicine, Endocrinology, Andrology and Metabolic Diseases Section, University of Bari Aldo Moro, 70124 Bari, Italy; giuseppina.biondi@uniba.it (G.B.); nicola.marrano@uniba.it (N.M.); annalisa.natalicchio@uniba.it (A.N.); 3 Department of Veterinary Medicine, University of Sassari, 07100 Sassari, Italy; acrovace@uniss.it

**Keywords:** bone marrow-derived canine mesenchymal stem cells, diabetes mellitus, dog, insulin-producing cells

## Abstract

**Simple Summary:**

Canine bone marrow-derived mesenchymal stem cells represent a potential starting source for the in vitro generation of functional insulin-producing cells that can be used in the clinical setting for treating diabetes mellitus type 1 in humans and dogs. Given the similar pathology and the close coexistence between humans and dogs, canine natural diabetes represents an excellent study model for translational research in human regenerative medicine. This work aims to generate glucose-responsive insulin-producing cells in vitro from canine bone marrow-derived mesenchymal stem cells using a two- and three-step protocol. Based on a single experiment, the three-step protocol proved effective in generating insulin-producing and secreting cells, but they were not responsive to glucose stimulation. Further experiments are needed to confirm this preliminary interesting result. In a clinical application perspective, a protocol capable of generating glucose-responsive insulin-producing cells is an indispensable feature.

**Abstract:**

Cell-based therapy using insulin-producing cells (IPCs) is anticipated as an alternative treatment option to insulin injection or pancreatic islet transplantation for the treatment of diabetes mellitus in both human and veterinary medicine. Several protocols were reported for the differentiation of mesenchymal stem cells (MSCs) into IPCs; to date, glucose-responsive IPCs have only been obtained from canine adipose tissue-derived MSCs (c*AD*-MSCs), but not from canine bone marrow-derived MSCs (cBM-MSCs). Therefore, this study aims to generate in vitro glucose-responsive IPCs from cBM-MSCs using two differentiation protocols: a two-step protocol using trichostatin (TSA) and a three-step protocol using mercaptoethanol to induce pancreatic and duodenal homeobox gene 1 (PDX-1) expression. A single experiment was carried out for each protocol. BM-MSCs from one dog were successfully cultured and expanded. Cells exposed to the two-step protocol appeared rarely grouped to form small clusters; gene expression analysis showed a slight increase in PDX-1 and insulin expression, but no insulin protein production nor secretion in the culture medium was detected either under basal conditions or following glucose stimulation. Conversely, cells exposed to the three-step protocol under a 3D culture system formed colony-like structures; insulin gene expression was upregulated compared to undifferentiated control and IPCs colonies secreted insulin in the culture medium, although insulin secretion was not enhanced by high-glucose culture conditions. The single experiment results suggest that the three-step differentiation protocol could generate IPCs from cBM-MSCs; however, further experiments are needed to confirm these data. The ability of IPCs from cBM- MSCs to produce insulin, described here for the first time, is a preliminary interesting result. Nevertheless, the IPCs’ unresponsiveness to glucose, if confirmed, would affect its clinical application. Further studies are necessary to establish a differentiation protocol in this perspective.

## 1. Introduction

Diabetes mellitus (DM) is one of the most common endocrinopathies in dogs, and the most commonly encountered clinical form is assimilated to DM type 1 (T1DM) in humans, which is caused by autoimmune pancreatic β-cell destruction with a failure to secrete insulin, subsequently leading to hyperglycemia [1]. Therapy for DM in dogs currently involves a symptomatic approach by administering lifelong exogenous insulin to control hyperglycemia and prevent associated complications, along with proper dietary management and exercise [1]. Given the similarity of the pathology and the close coexistence between humans and dogs, canine DM in natural conditions represents an excellent model of study for translational research in human regenerative medicine, which aims to replace β-cells and restore endogenous insulin secretion to achieve better glycemic control and avoid the complications associated with the pathology [2].

Stem cell therapy holds giant promise for the treatment of T1DM. In particular, mesenchymal stem cells (MSCs) provide a desirable substrate for producing insulin-producing cells (IPCs) that could be used in vivo in humans and dogs [3,4]. Pluripotent stem cells (embryonic stem cells, ESCs; induced pluripotent stem cells, IPSCs) have a high pancreatogenic capacity in vitro; however, due to ethical and safety issues related to their use, their clinical application is not feasible. In vitro differentiation protocols from canine adipose tissue-derived MSCs (c*AD*-MSCs) [3,4,5] and canine bone marrow (cBM-MSCs) [4,6] have recently been proposed. Until now, functional canine IPCs have been obtained from c*AD*-MSCs but not from cBM-MSCs [3].

Therefore, to date, a standard and effective protocol for the production of functional IPCs from cBM-MSCs in veterinary medicine is missing. This work aimed to generate glucose-responsive IPCs from cBM-MSCs in vitro, using two different differentiation protocols selected from the literature, with some modifications [5,7,8].

## 2. Materials and Methods

### 2.1. Retrieval and Expansion of cBM-MSCs

The cells used for this study derived from a frozen stock of cBM-MSCs previously isolated from a single canine donor [9] whose bone marrow specimen had been collected for therapeutic purposes at the Veterinary Clinic and Animal Production Section of the Department of Precision and Regenerative Medicine and Ionian Area—(DiMePRe-J) of the University of Bari Aldo Moro.

The cells, frozen in fetal bovine serum (FBS) with 10% dimethyl sulfoxide (DMSO), were subjected to rapid thawing at 37 °C in a thermostat and then centrifuged to remove the cryostatic medium. To proceed with cell expansion, cells were resuspended in Dulbecco’s modified Eagle’s medium (D-MEM) supplemented with 10% FBS, 1% of penicillin/streptomycin antibiotic solution and 1% of a 200 mM L- glutamine solution cultured at 37 °C in a 5% CO_2_ incubator. The medium was replaced every 3–4 days. Upon reaching 80–90% confluence, the cells were detached with trypsin-EDTA solution (Sigma-Aldrich, Burlington, MA, USA) and resuspended again until the required amount was obtained [10]. Here, the cells showed the appearance of fibroblast-like cells.

### 2.2. Characterization of cBM-MSCs

Obtained cells were characterized to ensure the presence of cBM-MSCs in the sample, which is the starting point for the in vitro induction protocol of IPCs. Three cell samples were initially thawed and cultured and sample 3 is the one used for the differentiation protocols. Therefore, specific surface antigens expressed by MSCs, which are part of the minimum criteria for the characterization of multipotent MSCs, were evaluated by RT-PCR analysis and normalized to RPL32 gene expression (*n* = 1) [11].

### 2.3. In Vitro Differentiation of cBM-MSCs into IPCs

The characterized cells were induced to form IPCs using two different protocols selected from the literature [5,7,8]. To this aim, the cBM-MSCs were seeded in conventional 6-well plates and cultured using a conventional culture system in the two-step protocol and a 3D culture system in the three-step protocol.

#### 2.3.1. Two-Step Protocol

**Figure 1:**
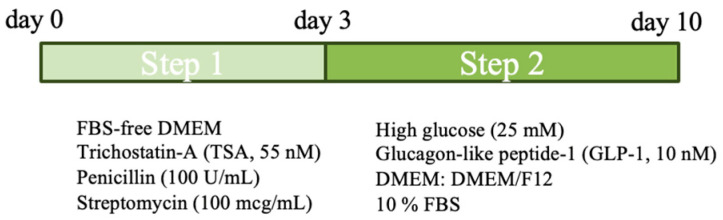
Schematic representation of the two-step protocol.

Differentiation was conducted according to the method previously reported [7,8], with some modifications. The protocol involved a total duration of 10 days divided into two steps (Figure 1). In the first step, cells were seeded in conventional 6-well plates (1 × 10^5^ cells/well) and cultured for 3 days in serum-free DMEM supplemented with 55 nM trichostatin-A (TSA) (Sigma-Aldrich), 100 U/mL penicillin and 100 mcg/mL streptomycin. In the second step, cells were cultured for additional 7 days in a high-glucose medium (25 mM) containing a 1:1 ratio of DMEM:DMEM/F12 (Sigma-Aldrich, Burlington, MA, USA). This mixture was supplemented with 10% FBS (Sigma-Aldrich, Burlington, MA, USA) and 10 nM glucagon-like peptide-1 (GLP-1, Sigma-Aldrich, Burlington, MA, USA). The culture medium was changed every 3 days. 

This protocol was conducted in a single experiment on 2 sets of cells (set 1 and set 2); a sample of cells not subjected to the protocol was provided for each set and used as a negative control.

#### 2.3.2. Three-Step Protocol

**Figure 2:**
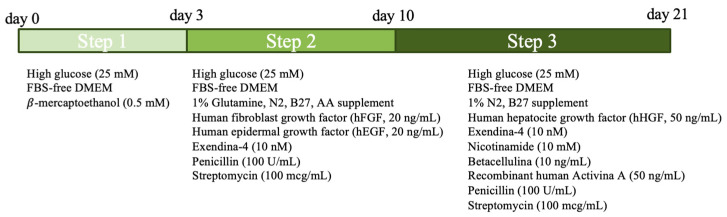
Schematic representation of the three-step protocol.

Differentiation was conducted using the protocol previously described by Teshima et al. [5], with some modifications (Figure 2). The cBM-MSCs were seeded in conventional 6-well plates (1 × 10^5^ cells/well) with a 3D culture system (Matrigel, New York, NY, USA). The protocol included a total duration of 21 days divided into 3 steps. A single experiment was carried out. In the first step (from day 0 to day 2), cBM-MSCs were cultured using a medium containing FBS-free DMEM with high glucose concentration (25 mM), 100 U/mL penicillin, 100 mcg/mL streptomycin and 0.5 mM β-mercaptoethanol (all reagents from Gibco, Waltham, MA, USA). In the second step (from day 3 to day 10), cells were cultured with a medium containing DMEM without FBS, with high glucose (25 mM), 100 U/mL penicillin, 100 mcg/mL streptomycin, 1% glutamine, 1% nonessential amino acids, 20 ng/mL recombinant human fibroblast growth factor (hFGF), 20 ng/mL recombinant human epidermal growth factor (hEGF), 1% N2 supplement, 1% B27 supplement and 10 nM Exendin-4 (hEGF and Exendin-4 from Sigma-Aldrich, all the other reagents from Gibco). Finally, in the third and final step (from day 11 to day 21), cells were cultured in a medium containing DMEM without FBS, with high glucose concentration (25 mM), 100 U/mL penicillin, 100 mcg/mL streptomycin, 1% N2 supplement, 1% B27 supplement, 10 nM Exendin-4, 10 mM Nicotinamide (Sigma-Aldrich, Burlington, MA, USA), 10 ng/mL Betacellulin (Sigma-Aldrich, Burlington, MA, USA), 50 ng/mL recombinant human Activin-A (Gibco, Waltham, MA, USA) and 50 ng/mL recombinant human hepatocyte growth factor (hHGF, Sigma-Aldrich, Burlington, MA, USA). In steps 2 and 3, the culture medium was replaced every 3 days.

This protocol was conducted on 1 set of cells; a sample of cells not subjected to the differentiation protocol was used as a negative control.

### 2.4. Morphological Analysis

Cells undergoing the differentiation protocol and negative controls were morphologically evaluated using a Nikon TMS Inverted Microscope (Nikon, Minato, Tokyo, Japan) at 10× and 20× magnification on days 3 and 10 for the two-step protocol and on days 8, 13, 16 and 21 for the three-step protocol. Images were captured using a Nikon Digital Sight Ds-fi1 Microscope C-mount Camera and the NIS-Element F3.0 software.

### 2.5. Gene Expression Analysis by RT-qPCR

Cells were lysed with RLT buffer supplemented with 1% β-mercaptoethanol. The lysate was homogenized in QIA shredder columns and treated with the RNeasy Mini Kit to extract total RNA, as per the protocol. All reagents were purchased from Qiagen, Hilden, Germany. To remove genomic DNA from the samples, DNase (RNase-Free DNase Set, Qiagen, Hilden, Germany) was used. The concentration of extracted RNA was determined by Fluorimetric Quantification using the Qubit (ThermoFisher Scientific, Waltham, MA, USA). An equal amount of RNA (1 μg) for all samples was back-transcribed into cDNA using the High Capacity DNA Reverse Transcription Kit (ThermoFisher Scientific, Waltham, MA, USA). A total of 25 ng of cDNA was used to amplify the fragments of interest in Real-Time PCR, using the ready-to-use master mix (iTaq Universal SYBR Green Supermix, Bio-Rad, Hercules, CA, USA). The CFX Connect Real-Time System (Bio-Rad, Hercules, CA, USA) thermal cycler was used for the assay. The reaction took place at the following amplification conditions: 95 °C for 2 min, followed by 40 cycles of 95 °C for 15 s and 60 °C for 60 s. Amplification of specific transcripts was confirmed by melting curve profiles at the end of each PCR. All reactions were performed in duplicate. Relative RNA levels were determined by analyzing the changes in SYBR green fluorescence during PCR using the 2^−ΔΔCt^ method. The mRNA level of each gene was normalized using glyceraldehyde-3-phosphate dehydrogenase (GAPDH) or ribosomal protein RPL32 as housekeeping. The primer sequences related to the genes of interest and housekeeping genes are shown in Table 1.

### 2.6. Analysis of Insulin Protein Expression by Immunofluorescence

To evaluate the presence of insulin, at the end of the differentiation protocol, the differentiated cells and negative controls were fixed in 4% paraformaldehyde, permeabilized with a 0.25% solution of Triton-X100 and blocked in 10% goat serum for 1 h in a humidified chamber at room temperature in order to saturate non-specific antibody sites. Subsequently, the cells were incubated overnight at 4 °C with a monoclonal antibody for insulin (1:200 dilution, ab181547 Abcam, Cambridge, UK) and then with an Alexa Fluor 488-conjugated secondary antibody (1:600 dilution, Thermo Fisher, Waltham, MA, USA) for 90 min in a dark chamber. After washing with 1 × PBS containing 0.05% Tween20, the cell nuclei were stained with DAPI. Immunofluorescence images were acquired on a Nikon ECLIPSE Ti-S fluorescence microscope (Nikon).

### 2.7. Glucose-Stimulated Insulin Secretion Assay

To verify the ability of the differentiated cells to assure a basal insulin secretion, at the end of the differentiation period, the levels of insulin released in the culture medium were measured using a canine insulin ELISA assay (Mercodia, Malmö, Sweden). To test the ability of the differentiated cells to secrete insulin in response to glucose, the differentiated cells were incubated for 90 min at 37 °C in HEPES-balanced Krebs-Ringer bicarbonate (KRBH) buffer (136 mM NaCl, 4.7 mM KCl, 1.25 mM CaCl_2_, 1.25 mM MgCl_2_, 5 mM KH_2_PO_4_, 25 mM NaHCO_3_, 10 mM HEPES and 0.5% BSA, pH 7.4) containing 3 mM glucose (all reagents are from Sigma-Aldrich, St. Louis, MO, USA). Subsequently, the cells were incubated for 40 min in KRBH containing low (3 mM) or high (25 mM) glucose concentrations or in a KCl solution (30 mM). Supernatants were collected at the end of each incubation period; insulin levels were measured using ELISA kits (Mercodia) and normalized against cellular total protein content.

### 2.8. Determination of Insulin Protein Content

To assess insulin protein content, cells were lysed on ice with lysis buffer (50 mM Trizma base, 1 mM EDTA, 1% Triton X 100, 150 mM NaCl, phosphatase inhibitor and protease inhibitor, all from Sigma-Aldrich). Protein concentration in cell lysates was determined by Bradford assay using Bio-Rad reagent; insulin content was assessed by ELISA (Mercodia) and normalized against total protein content.

## 3. Results

### 3.1. Characterization of cBM-MSCs

**Figure 3:**
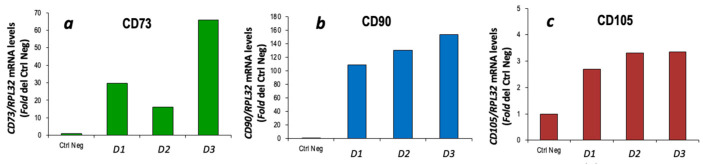
Characterization of cBM-MSCs. CD73 (**a**), CD90 (**b**) and CD105 (**c**) gene expression was evaluated by quantitative RT-PCR analysis and normalized to RPL32 gene expression (*n* = 1). Three cell samples were initially thawed and cultured and sample 3 is the one used for the differentiation protocols. Ctrl neg = cDNA from whole pancreas cells; D1 = cell sample dog 1; D2 = cell sample dog 2; D3 = cell sample dog 3.

CBM-MSCs derived from a single canine donor were successfully thawed, cultured and expanded as described in Section 2. The cultured cells showed an elongated, fibroblast-like morphology, arranged in a monolayer. The isolated cells expressed the CD73, CD90 and CD105 markers typical of MSCs (Figure 3). As sample 3 showed the higher expression of these markers, it was subsequently used in the two differentiation protocols.

### 3.2. In Vitro Differentiation of cBM-MSCs into IPCs

#### 3.2.1. Two-Step Protocol

**Figure 4:**
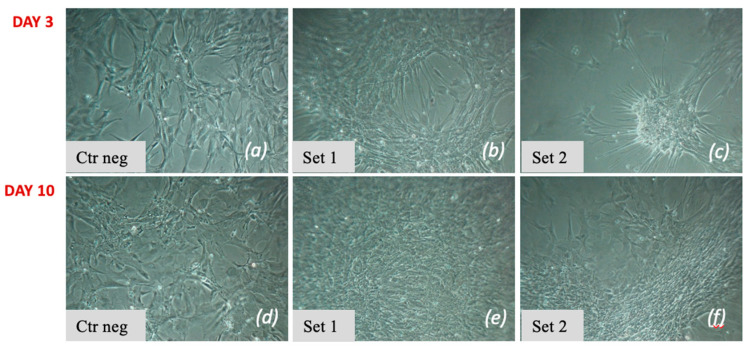
Morphological analysis of the cells subjected to the two-step protocol on day 3 (**a**–**c**) and day 10 (**d**–**f**), in relation to the negative control (**a**,**d**). On day 3, cells of set 1 (**b**) appeared similar to control cells; cells of set 2 (**c**) appeared grouped to form a small cluster. On day 10, cells of set 1 (**e**) and set 2 (**f**) proliferated and rarely tended to aggregate.

On day 3, the cells showed variable morphology between set 1 and set 2: in set 1 (Figure 4b), they appeared similar to control cells (Figure 4a), while in set 2 (Figure 4c), they appeared clustered to form small clusters of cells (a characteristic of differentiation). On day 10, the cells appeared more numerous, compared to the control condition (Figure 4d), and tended to form small clusters in some cell fields (Figure 4e,f).

**Figure 5:**
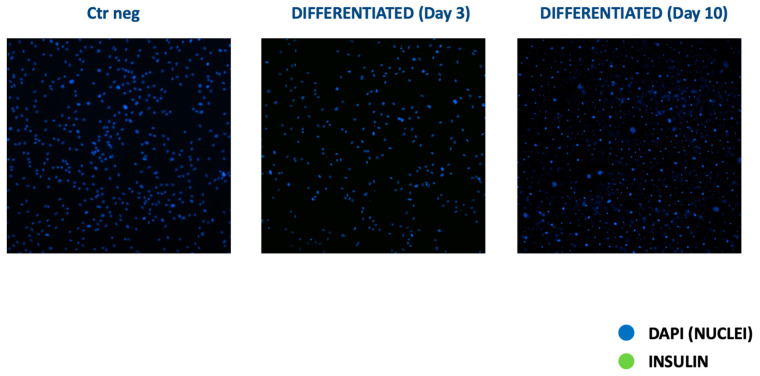
Analysis of insulin protein expression by immunofluorescence. Representative immunofluorescence images of cells subjected to the two-step protocol (only one set was analyzed). Sections were immunostained for insulin (green). Nuclear staining was performed with DAPI (blue). Magnification: 10× (*n* = 1).

Concordantly, immunofluorescence analysis revealed that the differentiated cells showed negative cytoplasmic staining for insulin, as did the undifferentiated cells (Figure 5).

Furthermore, no insulin secretion was detected either in the culture medium or following stimulation of the cells with KRBH solution containing glucose at low or high doses.

**Figure 6:**
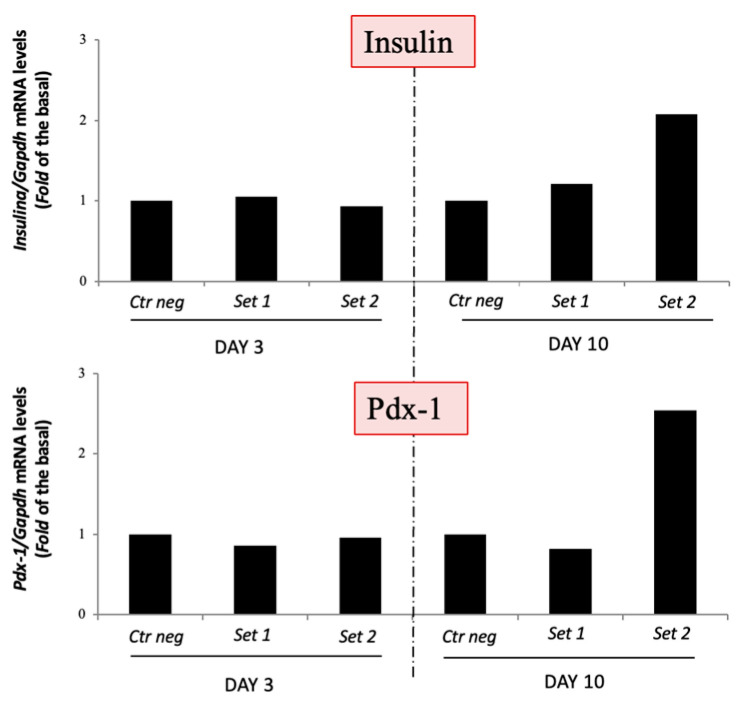
Analysis of insulin and PDX-1 gene expression in set 1 and set 2 on days 3 and 10. Insulin (**a**) and PDX-1 (**b**) gene expression was evaluated by quantitative RT-PCR analysis and normalized to GAPDH gene expression (*n* = 1).

Since the cells did not produce and secrete insulin, we looked at whether they at least expressed the insulin and PDX-1 genes and lost stemness. On day 3, mRNA analysis showed no increase in PDX-1 and insulin gene expression in set 1 and set 2 compared to the control cells, whereas on day 10, a slight increase in PDX-1 and insulin gene expression was appreciated in set 2 compared to the control cells (Figure 6).

**Figure 7:**
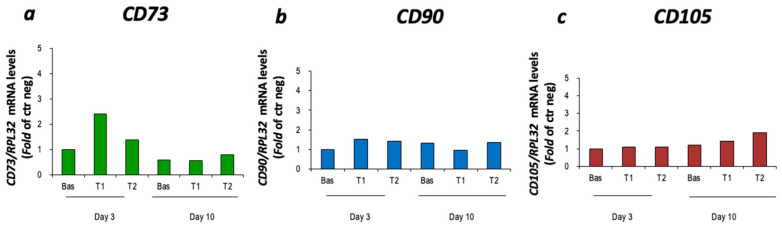
Comparison of stemness markers of cells subjected to the two-step differentiation protocol at day 3 and day 10. CD73 (**a**), CD90 (**b**) and CD105 (**c**) gene expression was evaluated by quantitative RT-PCR analysis and normalized to RPL32 gene expression (*n* = 1).

Nevertheless, characterized cBM-MSCs at the end of the two-step protocol showed no reduction in stemness markers compared to baseline conditions (Figure 7).

#### 3.2.2. Three-Step Protocol

**Figure 8:**
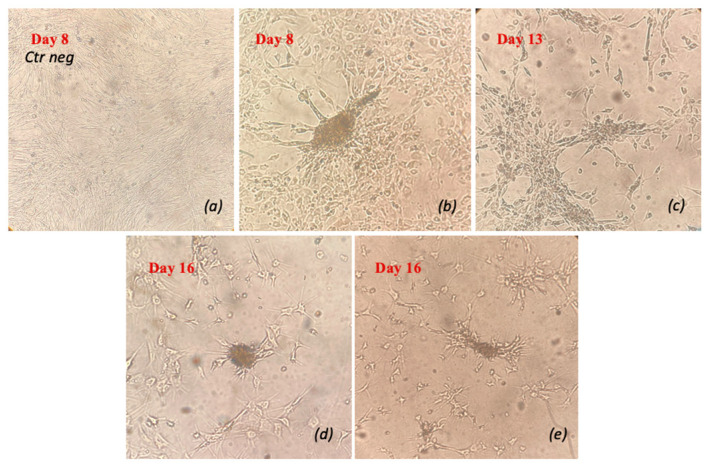
Morphological analysis of the cells subjected to the three-step protocol on day 8 (**b**), day 13 (**c**) and day 16 (**d,e**) in relation to the negative control on day 8 (**a**). (*n* = 1).

Cells subjected to the three-step differentiation protocol in a 3D culture system (Matrigel) formed colony-like structures starting from day 8 of differentiation, without showing any further proliferation compared to the basal condition (Figure 8).

**Figure 9:**
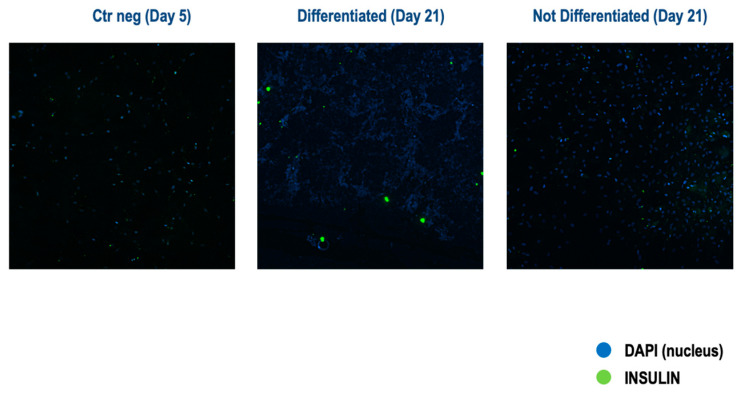
Analysis of insulin protein expression by immunofluorescence. Representative immunofluorescence images of cells subjected to the three-step protocol. Sections were immunostained for insulin (green). Nuclear staining was with DAPI (blue). Magnification: 10× (*n* = 1).

Concordantly, immunofluorescence analysis revealed that differentiated cells showed positive cytoplasmic staining for insulin on day 21, while undifferentiated cells on day 21 and basal cells on day 5 were negative for insulin (Figure 9). Of note, as shown by immunofluorescence images, not all cells were positive for insulin staining, suggesting that only some MSCs underwent the differentiation process.

**Figure 10:**
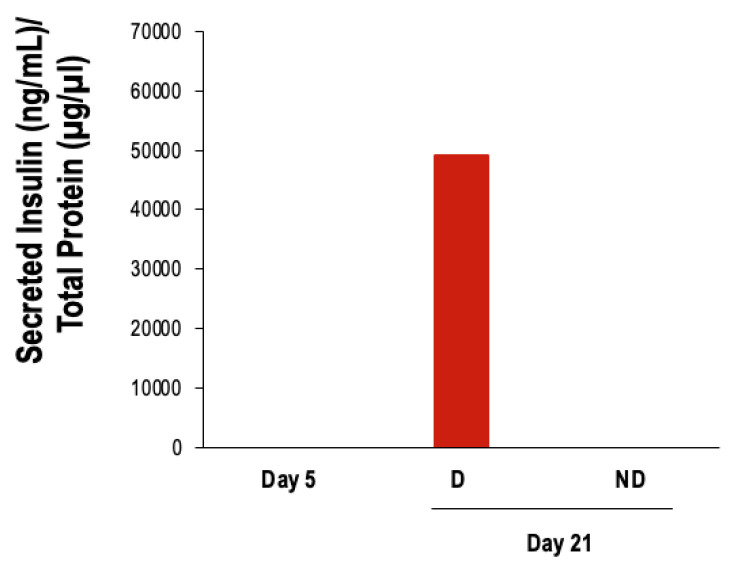
Analysis of secreted insulin levels in the culture medium at day 21 (*n* = 1). D: cells subjected to the differentiation protocol; ND: cells not subjected to the differentiation protocol.

Furthermore, cells differentiated by three-step protocol secreted insulin into the culture media at day 21, unlike the negative control (Figure 10).

**Figure 11:**
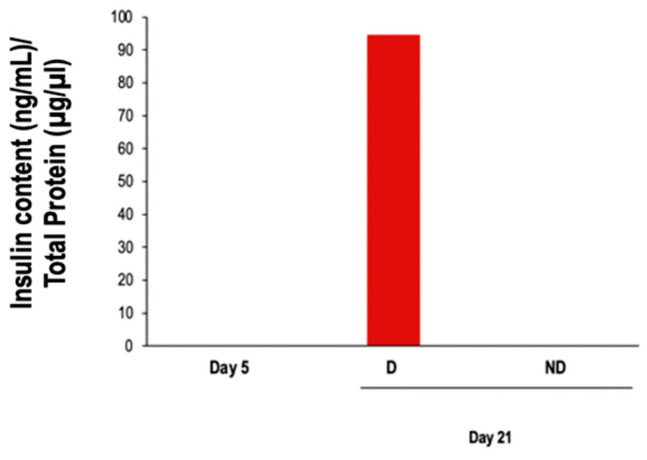
Analysis of insulin protein expression at day 21 (*n* = 1). D: cells subjected to the differentiation protocol; ND: cells not subjected to the differentiation protocol.

Interestingly, insulin protein expression was upregulated in differentiated cells on day 21 compared to undifferentiated control on day 21 and compared to basal condition on day 5 (Figure 11).

**Figure 12:**
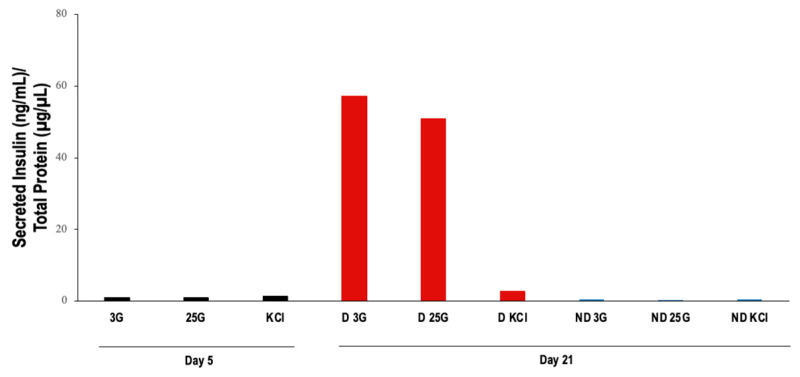
Glucose-stimulated insulin secretion assay. 3G: stimulation with 3 mM glucose; 25G: stimulation with 25 mM glucose; KCl: stimulation with KCl; (*n* = 1). D: cells subjected to differentiation protocol; ND: cells not subjected to differentiation protocol.

However, insulin secretion obtained by incubating the cells in KRBH solution containing low levels of glucose (3 mM) was not increased by stimulation with high glucose (25 mM) or KCl (Figure 12).

**Figure 13:**
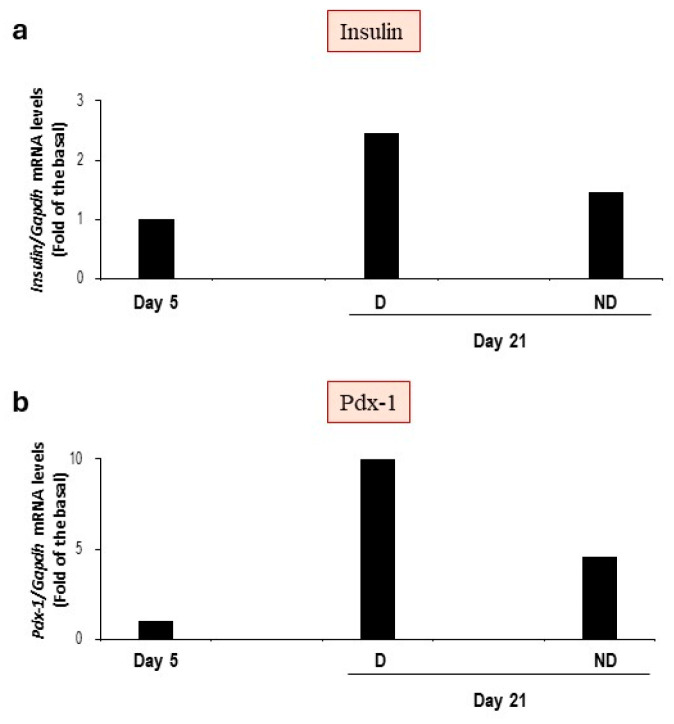
Analysis of insulin and PDX-1 gene expression in cells subjected to the three-step protocol, on days 5 and 21. Insulin (**a**) and PDX-1 (**b**) gene expression was evaluated by quantitative RT-PCR analysis and normalized to GAPDH gene expression (*n* = 1). D: cells subjected to the differentiation protocol; ND: cells not subjected to the differentiation protocol.

**Figure 14:**
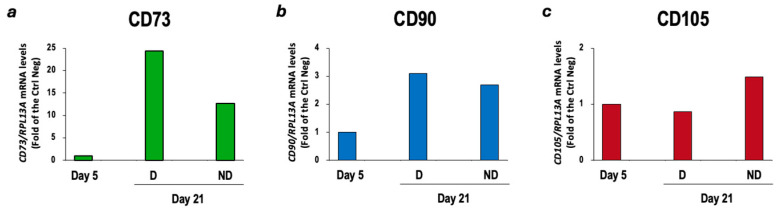
Comparison of stemness markers of cells subjected to the three-step differentiation protocol at day 5 and day 21. CD73 (**a**), CD90 (**b**) and CD105 (**c**) gene expression was evaluated by quantitative RT-PCR analysis and normalized to RPL13A gene expression (*n* = 1). D: cells subjected to the differentiation protocol; ND: cells not subjected to the differentiation protocol.

Finally, on day 21, a slight increase in insulin and PDX-1 gene expression was appreciated in differentiated cells compared to cells subjected to the protocol at day 5 and undifferentiated cells (Figure 13). In addition, at the end of the three-step protocol, the cells subjected to differentiation protocol still express stemness markers (Figure 14). These results further suggest that only some MSCs underwent the differentiation process.

## 4. Discussion

In the last decade, especially in human medicine, several studies have been conducted for the in vitro differentiation of MSCs into IPCs [8,12,13], as they can be an excellent alternative treatment to pancreatic islet transplantation.

Recently, the attention of researchers has turned to dogs, which are considered the ideal translational animal spontaneous model for the treatment of T1DM in humans. Therefore, the use of IPCs could be a viable alternative option for the treatment of diabetes in humans and dogs.

MSCs can be obtained from different adult tissues such as bone marrow (BM) and adipose tissue (*AD*). In humans, BM-derived MSCs have a higher capacity to differentiate into IPCs than those obtained from subcutaneous adipose tissue [14], as well as possessing better proliferative and differentiative capacities [15].

Only recently, differentiation protocols starting from canine MSCs, derived either from BM (cBM-MSCs) [4,6,16] or from *AD* (c*AD*-MSCs) [3,5], have been investigated. However, there is still no effective protocol to produce functional IPCs that are capable of secreting insulin in response to glucose stimulation, which would make them suitable for clinical application in diabetic patients.

For our experiments we choose two different protocols previously reported in the literature.

The first protocol was a two-step protocol that was effective in generating poorly glucose-responsive IPCs in humans [8,13] and mice [7]. The second protocol was a three-step protocol that was effective in producing partly glucose-responsive IPCs starting from c*AD*-MSCs [5]. The initial step in IPCs differentiation is to induce PDX-1 expression. PDX-1 is one of the well-known transcription factors that are fundamental for *β* -cell development and function [17]. PDX-1 regulates pancreatic islet maturation by stimulating insulin and other downstream gene expression. In the present study, we used trichostatin (TSA) in the two-step protocol and *β* -mercaptoethanol in the three-step protocol to induce PDX-1 expression.

The reprogramming of BM cells into IPCs may depend on chromatin modulation. TSA, a histone deacetylase inhibitor (HDAC), has been shown to have the ability to remodel chromatin, allowing increased access to transcription factors. Furthermore, it is a potent driver of pancreatic cell line progenitors and can enable the differentiation of BM cells into IPCs in the presence of high glucose concentrations and GLP-1 [7,18]. Glucose is a growth factor for *β*-cells [17,19], as it promotes *β* -cell replication in vitro and in vivo at concentrations of 20–30 mM [20]. In contrast, GLP-1 is a hormone capable of converting intestinal epithelial cells into functional IPCs [21]. With this work, we report for the first time the use of a TSA-based protocol starting from cBM-MSCs, which, in our single experiment, did not prove effective in generating IPCs. In fact, mild modifications of the cells were observed at the morphological level, and small clusters of cells were observed only in some fields, retaining a high replicative rate, which is indicative of poor differentiation. Cells subjected to the protocol showed a slight increase in PDX-1 and insulin gene expression compared to negative controls but did not show insulin protein expression or secretion. In light of the poor results obtained, a retrospective comparison of the stemness markers of the pre- and post-differentiation cells was performed, from which no reduction in these markers emerged. This supports and confirms the lack of differentiation of these cells. The reasons for this could be in the short duration of the protocol, the scarcity of extrinsic factors used, the two-dimensional culture system or the variability in an individual donor or species.

The three-step protocol was previously proposed starting from c*AD*-MSCs and was found to be effective in producing poor glucose-responsive IPCs [5]. In the first step of the same protocol, *β* -mercaptoethanol was added to reach the expression of the transcription factor PDX-1, which is required for early embryonic pancreatic development and subsequent differentiation [22,23]. In the second step, EGF and FGF were used, as these factors have been shown to stimulate proinsulin biosynthesis in an experimental model [24] and play an important role in cell proliferation, differentiation and survival [19,25]. Exendin-4 can increase the expression of β-cell-related genes PDX-1, Nkx2.2, Isl-1 and MafA [26]. The third step involved the use of activin-A and betacellulin; the combination of these two factors increases the proliferation of IPCs [27,28]. Nicotinamide is an effective inducer used to preserve islet viability and function through the inhibition of poly-ADP-ribose polymerase (PARP) [29,30]. It has been suggested that HGF is an important regulator of β-cell function and proliferation and that its effects are improved by activin-A [31,32]. High concentrations of glucose in the culture medium were used throughout the differentiation protocol, as high glucose levels represent a potent inducer for pancreatic islet differentiation [17,19]. For the differentiation of IPCs, Matrigel was used for the three-dimensional cell culture. The 3D cell culture system supports tissue-specific function and physiological cell–cell and cell–matrix interactions. These factors provide several benefits by increasing cell viability, improving cell type-specific function and gene expression, and increasing cellular secretion of proteins [33,34]. In the study by Teshima et al. [5], IPCs generated in the 3D culture system showed increased insulin secretion compared to cells grown in conventional 2D culture dishes. These results are in agreement with a recent study showing that the generation of IPCs from cBM-MSCs requires a 3D culture condition [4]. The preliminary results obtained from the present study show that cBM-MSCs can be differentiated into IPCs in a 3D culture system using the three-step protocol previously described by Teshima [5]. However, IPCs obtained with this protocol from cBM-MSCs can produce insulin and secrete it in the culture medium under basal conditions but not in response to glucose stimulation. The lack of response to glucose observed at the end of differentiation could be related to the numerous variables of the protocol used; it could also depend on the intrinsic differentiative and proliferative potential of BM-MSCs in the canine species. In addition, it should be recalled that only some BM-MSCs underwent the differentiation process. Although no differences between hBM-MSCs and h*AD*-MSCs have been reported in humans [35], a recent work by Rodprasert et al. [4] assessed that the differentiation potential of BM-MSCs and *AD*-MSCs does not overlap in dogs. However, no protocol has yet been developed that is effective in producing large quantities of glucose-responsive IPCs, regardless of the starting tissue. The limitation of this study is that it is based on a single experiment for each protocol; thus, further experiments are needed to confirm results. Nevertheless, the capability of BM-MSCs to secrete insulin has to be considered an interesting result, which is described here for the first time.

The final aim of in vitro studies for IPCs generation is the clinical applicability for the management of diabetes in natural conditions. The cells’ responsiveness to glucose concentrations, sterile authorized environments for production, the choice of the most suitable route of administration, the longevity of the cells post-implantation and their protection from autoimmune processes are only some of the challenges to be faced [12,36].

## 5. Conclusions

The results of this preliminary study based on a single experiment demonstrate that the selected three-step differentiation protocol could generate IPCs in a 3D culture system from canine BM-MSCs, although with limited functional properties. Further experiments are needed to confirm these results and further studies are needed to establish an effective protocol capable of differentiating cBM-MSCs into glucose-responsive IPCs, a feature indispensable for a clinical application perspective. This preliminary study further suggests the importance of valid cross-sectional studies of comparative medicine in order to find viable innovative options for the treatment of spontaneous diabetes in dogs and in humans.

## Figures and Tables

**Table 1:** Sequences of the primers used for RT-qPCR.

Target	Forward Primer (5′-3′)	Reverse Primer (5′-3′)
Insulin	AGGGAGGTGGAGGACCTG	CAGCACTGCTCCACGATG
RPL13A	GCCGGAAGGTTGTAGTCGT	GGAGGAAGGCCAGGTAATTC
RPL32	TGGTTACAGGAGCAACAAGAAA	GCACATCAGCAGCACTTCA
PDX-1	GACAACAGGAACTACAAGTCGGAAT	TGTTTCGGGACAGATGAAGGT
CD73	GCCGGAAGGTTGTAGTCGT	GGAGGAAGGCCAGGTAATTC
CD90	TGGTTACAGGAGCAACAAGAAA	GCACATCAGCAGCACTTCA
CD105	GACAACAGGAACTACAAGTCGGAAT	TGTTTCGGGACAGATGAAGGT
GAPDH	CGAGATCCCGCCAACATCAA	CTCCATGGTGGTGAAGACCC

## Data Availability

The original dataset is available from the authors.
